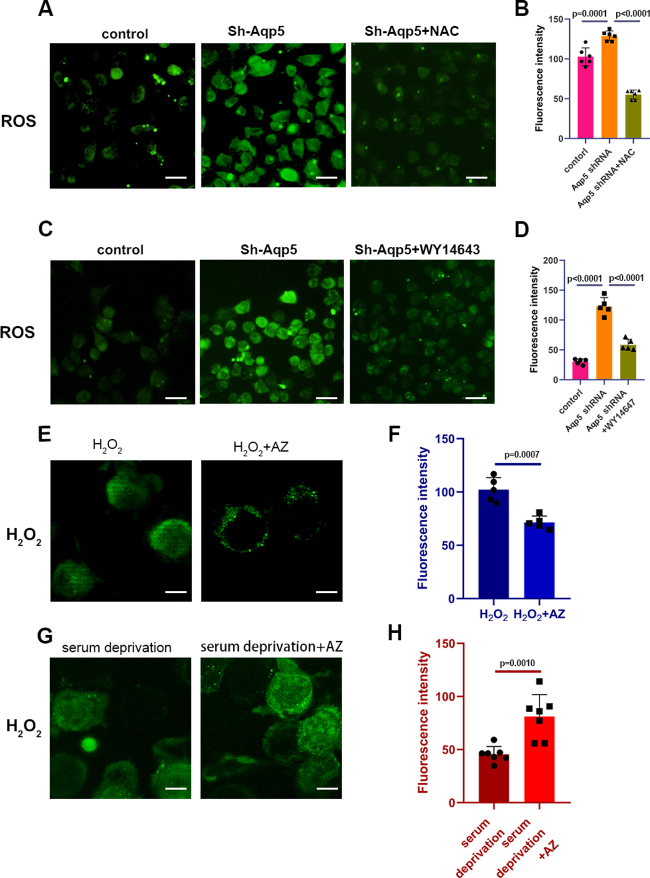# Aquaporin five deficiency suppresses fatty acid oxidation and delays liver regeneration through the transcription factor PPAR

**DOI:** 10.1016/j.jbc.2025.108303

**Published:** 2025-02-11

**Authors:** Bin Li, Shixu Liu, Wenshuo Han, Peirong Song, Hetong Sun, Xin Cao, Guohu Di, Peng Chen

**Affiliations:** 1 School of Basic Medicine, Qingdao University, Qingdao, Shandong Province, China; 2 Institute of Stem Cell Regeneration Medicine, School of Basic Medicine, Qingdao University, Qingdao, Shandong Province, China; 3 Department of Ophthalmology, Qingdao Eighth People's Hospital, Qingdao, Shandong Province, China

**Keywords:** Aqp5, liver regeneration, hydrogen peroxide, lipid accumulation, PPAR pathway

## Abstract

After 70% partial hepatectomy (PHx), the metabolic pathways leading to hepatocyte lipid droplet accumulation during liver regeneration remain unclear. Aquaporin 5 (Aqp5) is an aquaporin that facilitates the transport of both water and hydrogen peroxide (H_2_O_2_). In this study, we observed delayed liver regeneration following PHx in *Aqp5* knockout (*Aqp5*^*−/−*^) mice. Considering the role of Aqp5 in H_2_O_2_ transport, we hypothesized that deficiency in *Aqp5* may induce oxidative stress and hepatocyte injury. Through the measurement of reactive oxygen species (ROS) and redox-related indices, we observed significant alterations in ROS levels as well as malondialdehyde (MDA), superoxide dismutase (SOD), and reduced glutathione (GSH) concentrations in regenerating livers lacking *Aqp5* compared to wild-type controls. Oil Red O and 4-hydroxynonenal (4-HNE) staining results indicated that *Aqp5* deficiency caused lipid accumulation during liver regeneration. The transcriptome sequencing results showed that the PPAR pathway is inhibited during the liver regeneration process in *Aqp5* gene-knockout mice. The administration of the WY-14643 agonist, which targets the PPAR pathway, significantly mitigated delayed liver regeneration by enhancing hepatocyte proliferation and reducing lipid accumulation caused by *Aqp5* deficiency. Our findings highlight the crucial role of *Aqp5* in regulating H_2_O_2_ levels and lipid metabolism through the PPAR pathway during liver regeneration.

The liver exhibits a substantial capacity for regeneration, which encompasses an intricate cascade of various cytokines, growth factors, and metabolic modifications (1, 2). The 70% partial hepatectomy (PHx) model is predominantly employed in investigating liver regeneration (3). After PHx, the residual hepatocytes swiftly enter the cell cycle and undergo rapid proliferation to compensate for the body's energy demands (4).

After PHx, the liver undergoes a series of metabolic changes, including temporary hepatic lipid accumulation or transient regeneration-related steatosis (TRAS). This phenomenon is considered one of the most significant physiological changes observed during PHx-induced liver regeneration and is essential for proper physiological liver regeneration (5). During TRAS, accumulated lipids originate from systemic lipolysis of peripheral fat storage and are rapidly utilized for ATP production through β-oxidation to facilitate efficient liver regeneration (5, 6, 7). In murine models, levels of TRAS typically peak at 24 h and subsequently decline to low levels between 48 to 72 h after PHx (8, 9). Although excessive lipid accumulation during liver regeneration can have detrimental effects on hepatocyte proliferation, the underlying mechanisms governing lipid accumulation and metabolism remain elusive (10, 11).

Hydrogen peroxide (H_2_O_2_) is generated during rapid liver regeneration in response to stimulation from growth factors, chemokines, and environmental stressors (12, 13, 14, 15). However, excessive production of H_2_O_2_ in hepatocytes can lead to oxidative stress and subsequent hepatocyte death (13, 16). Aquaporins (Aqps) are transmembrane proteins responsible for facilitating water transport across cell membranes (17). Certain isoforms of the Aqps family have been demonstrated to enhance the transport of H_2_O_2_ across biological membranes. These specific Aqps, referred to as peroxide proteins, play a vital role in regulating redox signaling in various organisms (17, 18). Aberrant expression of Aqps in the gastrointestinal system has been associated with the pathogenesis and progression of various diseases (19). In mammalian livers, multiple members of the aquaporin subfamily, namely Aqp1, Aqp4, Aqp5, Aqp8, and Aqp9, are present. Among these isoforms, both Aqp5 and Aqp9 function as water channels facilitating efficient water transport while also serving as peroxide porins enabling effective H_2_O_2_ transportation (17, 18, 20, 21). The present study demonstrates that *Aqp5* is expressed in both mouse and human liver cells, as well as the hepatoblastoma cell line HepG2.2.15 and the HCC cell line Huh7, indicating a close association with *Aqp5* (22, 23, 24, 25).

Peroxisome proliferator-activated receptors (PPARs) are members of a superfamily of intranuclear receptor transcription factors that regulate gene expression (26). PPARs consist of three subtypes: PPARα, PPARβ, and PPARγ, each subtype regulates distinct target genes (27). Known as fatty acid receptors primarily involved in the regulation of fatty acid metabolism, PPARα is highly expressed in the liver and plays a crucial role in hepatic lipid metabolism (28, 29, 30, 31). The PPARα gene plays a significant role in hepatic lipid development, storage, and transport as well as fatty acid oxidation (FAO) (32). It exerts control over the expression of genes associated with FAO in the liver, primarily regulating the mitochondrial β-oxidation system, peroxisomal β-oxidation system, and microsomal ω-oxidation system (33). Additionally, PPARα modulates the expression of key enzymes involved in the peroxisomal β-oxidation pathway, such as acetyl coenzyme-A oxidase, ACSL1, dehydrogenase multifunctional enzyme, and ketoacetyl coenzyme-A thiolase (34).

The ACSL is classified into five subfamilies based on the length of the acyl chains, namely ACSL1, ACSL3, ACSL4, ACSL5, and ACSL6 (35). The predominant isoform in the liver, ACSL1, plays a crucial role in hepatic lipid metabolism and serves as a target gene for PPAR (36). In mammals, intracellular processing of long-chain fatty acids requires their initial activation of acyl-CoA by ACSL. Primarily located in the cytoplasm, ACSL1 esterifies long-chain acyl-CoA which subsequently undergoes β-oxidation (37). Additionally, it plays a crucial role in the synthesis of long acyl-CoA esters necessary for the regulation of diverse physiological processes. Dysregulation of ACSL1 as a provider of substrates in anabolic or catabolic pathways can result in disorders such as hepatic steatosis, hyperacidemia, insulin resistance, and others (37). Furthermore, Cytochrome P450 monooxygenase (Cyp4a), being another PPAR-sensitive target gene, is responsible for microsomal fatty acid oxidation (38). In mouse livers, Cyp4a10, Cyp4a12a, Cyp4a12b, and Cyp4a14 belong to the Cyp4a subfamily and play pivotal roles in maintaining lipid homeostasis and regulating fatty acid signaling pathways (39, 40). Studies conducted on PPARα^−/−^ mice have demonstrated that hepatic expression of the Cyp4a genes is significantly diminished in the absence of PPARα (Cyp4a10, Cyp4a12, and Cyp4a14 in mice; Cyp11 in humans) (41).

In this study, it was demonstrated that *Aqp5* plays a pivotal role in liver regeneration by regulating oxidative stress through H_2_O_2_ concentration. The regenerated livers of *Aqp5*^*−/−*^ mice exhibited accumulated lipid droplets, a significant downregulation of the PPAR pathway, and impaired hepatocyte proliferation due to excessive lipid droplet accumulation. Furthermore, the activation of the PPAR pathway using WY-14643 promoted hepatocyte proliferation. Therefore, *Aqp5* may facilitate liver regeneration through its involvement in fatty acid oxidation *via* the PPAR pathway.

## Results

### Localization of Aqp5 expression and lipid alterations in the liver of *Aqp5*^*−/−*^ mice

**Figure 1:**
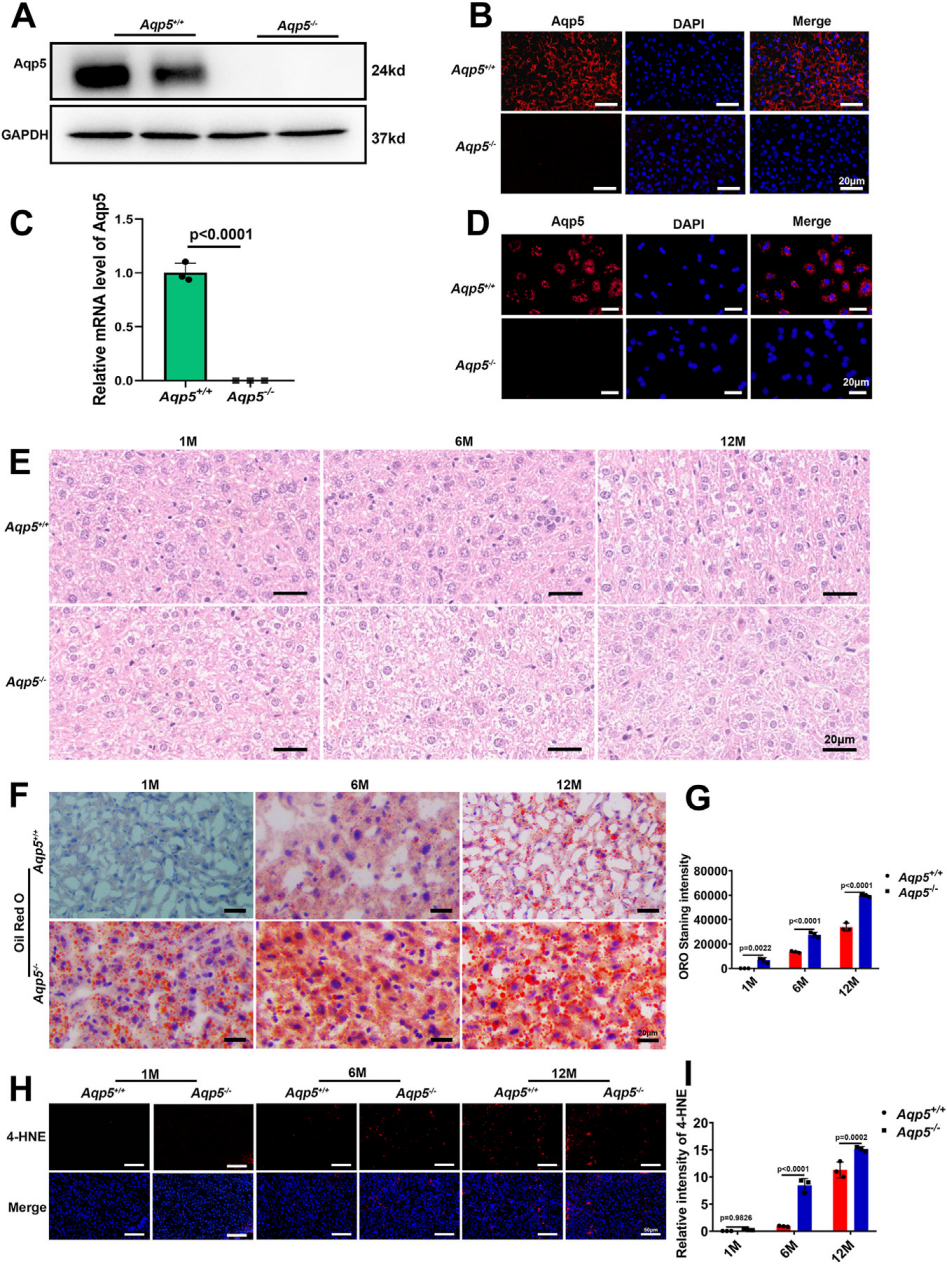
**Aqp5 expression localization and lipid changes in the liver.***A*, Western blotting showed the expression level of *Aqp5* and GAPDH in hepatic tissues. *B*, immunofluorescence staining showed the expression of Aqp5 in hepatic tissues (*red*, *Aqp5*; *blue*, 4 6-diamidine-2-benzene index [DAPI]). *C*, the expression of Aqp5 mRNA in the hepatic tissues was determined by qPCR (n = 3). *D*, the expression of Aqp5 in primary hepatocytes was detected by immunofluorescence staining. *E,* H&E staining of hepatic tissues. *F*, ORO staining of hepatic tissues. *G*, ORO staining intensity was analyzed by ImageJ software. Statistics were calculated by using Two-way ANOVA with Sidak's multiple comparison test. For a 2-way ANOVA comparing time points (1, 6, 12 months) and genotype (wild-type *versus* knockout) for their effect on ORO Staining intensity: Time: F (2, 12) = 839.5, *p* < 0.0001; Genotype: F (1, 12) = 317.8, *p* < 0.0001; Interaction (Time × Genotype): F (2, 12) = 40.57, *p* < 0.0001. *H*, 4-HNE staining in *Aqp5*^*−/−*^ and *Aqp5*^*+/+*^ mice liver. *I*, 4-HNE staining intensity analyzed by ImageJ software. Statistics were calculated by using Two-way ANOVA with Sidak's multiple comparison test. For a 2-way ANOVA comparing time points (1, 6, 12 months) and genotype (wild-type *versus* knockout) for their effect on relative intensity of 4-HNE: Time: F (2, 12) = 401.3, *p* < 0.0001; Genotype: F (1, 12) = 102.5, *p* < 0.0001; Interaction (Time × Genotype): F (2, 12) = 30.37, *p* < 0.0001. Data were shown as mean ± SD. Scale bar: (*B*, *D*–*F*) 20 μm. (*H*) 50 μm.

**Supplementary Fig1:**
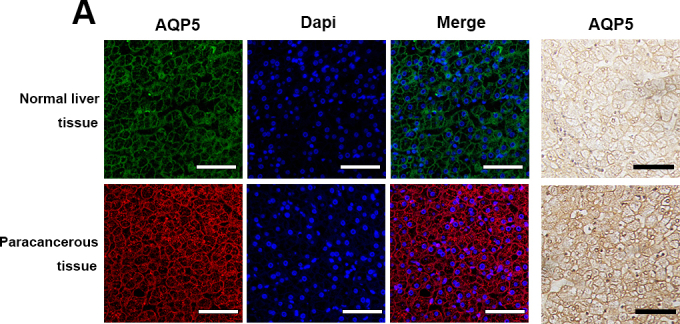


**Supplementary Fig2:**
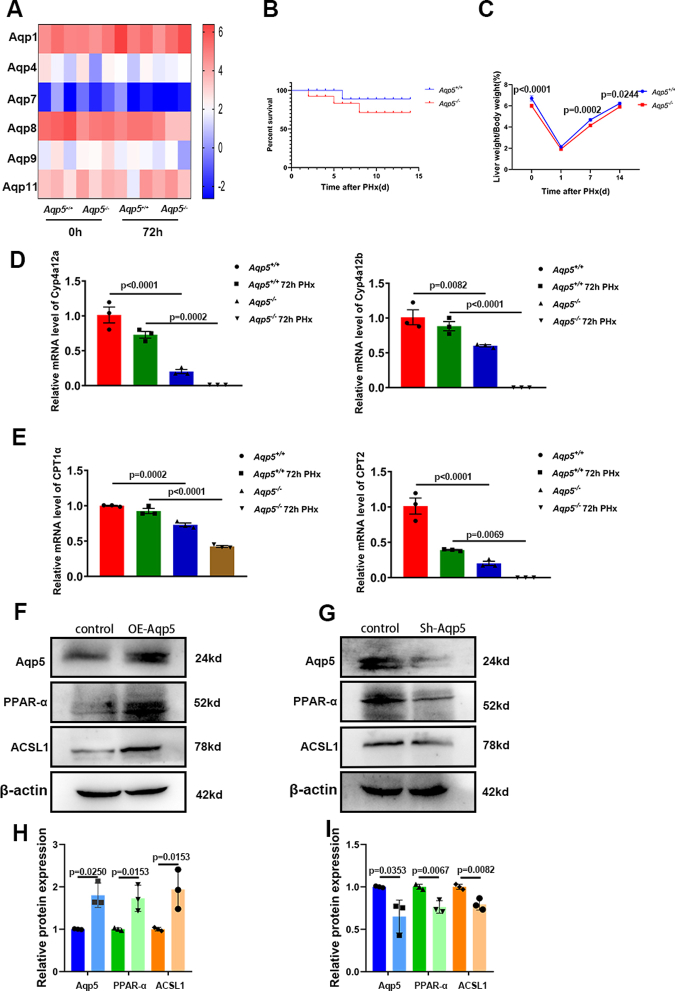


Western blot analysis was conducted on hepatic tissues obtained from wild-type (*Aqp5*^*+/+*^) and *Aqp5*^*−/−*^ mice. Aqp5 expression was detected in the hepatic tissues of *Aqp5*^*+/+*^ mice, whereas it was absent in *Aqp5*^*−/−*^ mice (Fig. 1*A*). Consistent results were obtained from RT-qPCR analysis of hepatic tissues from both *Aqp5*^*+/+*^ and *Aqp5*^*−/−*^ mice (Fig. 1*C*). Immunofluorescence staining confirmed these findings by demonstrating the presence of Aqp5 in the hepatic tissues and primary hepatocyte of *Aqp5*^*+/+*^ mice, but its absence in those of *Aqp5*^*−/−*^ mice (Fig. 1, *B* and *D*). We further investigated the expression of AQP5 in normal liver tissues and paracancerous tissues from human samples (Fig. S1*A*). As well, the expression of other AQPs was examined in the liver of *Aqp5*^*+/+*^ and *Aqp5*^*−/−*^ mice (Fig. S2*A*). H&E staining revealed hepatocytes with enlarged size and disorganized structures in 12-month-old *Aqp5*^*−/−*^ mice (Fig. 1*E*), whereas ORO staining demonstrated accumulation of neutral triglycerides within their hepatocytes (Fig. 1*F*). The intensity of neutral triglycerides exhibited a significant increase from ages one to 12 months, surpassing the levels observed in age-matched wild-type controls (n = 3 per group) (Fig. 1*G*). Moreover, there was an evident accumulation of lipid peroxidation end-products within the hepatocytes of aged *Aqp5*^*−/−*^ mice, as indicated by positive staining with anti-4-HNE antibodies; this effect became more pronounced with advancing age compared to *Aqp5*^*+/+*^ mice over time (n = 3 per group) (Fig. 1, *H* and *I*).

### The deficiency of *Aqp5* resulted in decreased hepatocyte proliferation during liver regeneration following PHx

**Figure 2:**
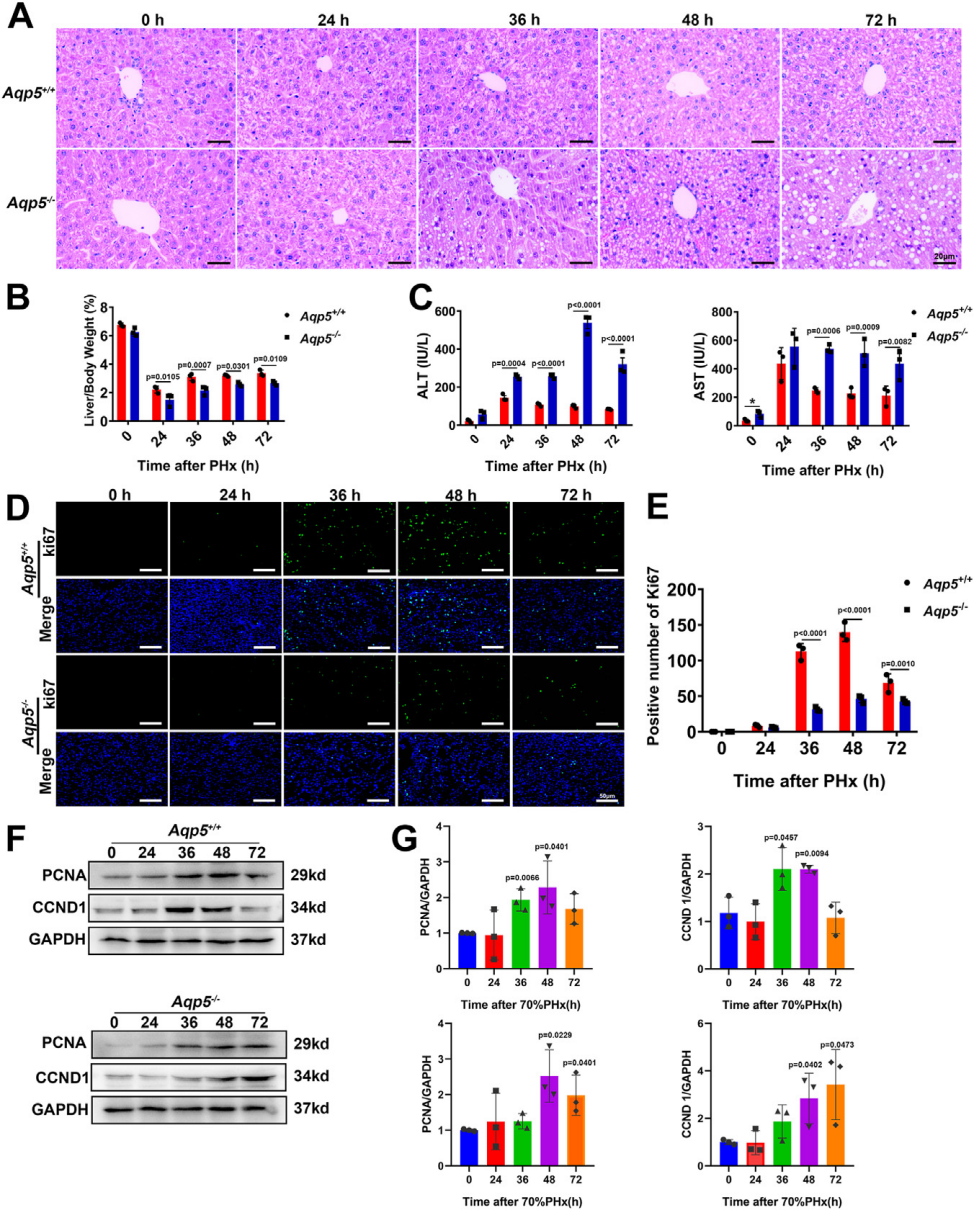
***Aqp5* deficiency resulted in decreased hepatocyte proliferation during liver regeneration after PHx.***A*, H&E staining was performed at indicated time points after PHx. *B*, Liver-to-body weight ratios were calculated at indicated time points. Statistics were calculated by using Two-way ANOVA with Sidak's multiple comparison test. For a 2-way ANOVA comparing time points (0, 24, 36, 48, 72 h) and genotype (wild-type *versus* knockout) for their effect on Liver/Body Weight: Time: F (4, 20) = 361.0, *p* < 0.0001; Genotype: F (1, 20) = 59.66, *p* < 0.0001; Interaction (Time × Genotype): F (4, 20) = 0.6463, *p* = 0.6360. *C*, the activity of ALT and AST was measured in the serum of *Aqp5*^*−/−*^ and *Aqp5*^*+/+*^ mice at indicated time points after PHx. Statistics were calculated by using Two-way ANOVA with Sidak's multiple comparison test. For a 2-way ANOVA comparing time points (0, 24, 36, 48, and 72 h) and genotype (wild-type *versus* knockout) for their effect on ALT: Time: F (4, 20) = 78.09, *p* < 0.0001; Genotype: F (1, 20) = 374.3, *p* < 0.0001; Interaction (Time × Genotype): F (4, 20) = 47.71, *p* < 0.0001. For a 2-way ANOVA comparing time points (0, 24, 36, 48, 72 h) and genotype (wild-type vs. knockout) for their effect on AST: Time: F (4, 20) = 28.01, *p* < 0.0001; Genotype: F (1, 20) = 48.90, *p* < 0.0001; Interaction (Time × Genotype): F (4, 20) = 2.993, *p* = 0.0435. *D*, immunofluorescence of Ki67 at indicated time points after PHx was performed. *E*, Ki67 positive hepatocytes were calculated. Statistics were calculated by using Two-way ANOVA with Sidak's multiple comparison test. For a 2-way ANOVA comparing time points (0, 24, 36, 48, 72 h) and genotype (wild-type *versus* knockout) for their effect on positive number of Ki67: Time: F (4, 20) = 195.5, *p* < 0.0001; Genotype: F (1, 20) = 246.4, *p* < 0.0001; Interaction (Time × Genotype): F (4, 20) = 57.86, *p* < 0.0001. *F*, Western blotting of PCNA and CCND1 at indicated time points after PHx was performed. GAPDH was used as a loading control. *G*, PCNA and CCND1 expression was quantified (n = 3). Statistics were calculated by using a One-way ANOVA with Sidak's multiple comparisons. The PCNA results of *Aqp5*^*+/+*^ mice showed that the overall difference is significant, with F (4, 10) = 4.976, *p* = 0.0181. The CCND1 results of *Aqp5*^*+/+*^ mice showed that the overall difference is significant, with F (4, 10) = 8.277, *p* = 0.0033. The PCNA results of *Aqp5*^*−/−*^ mice showed that the overall difference is significant, with F (4, 10) = 3.931, *p* = 0.0360. The CCND1 results of *Aqp5*^*−/−*^ mice showed that the overall difference is significant, with F (4, 10) = 5.339, *p* = 0.0145. Data were shown as mean ± SD. Scale bar: (*A*) 20 μm. (*D*) 50 μm.

To investigate the role of *Aqp5* in liver regeneration, we conducted 70% PHx on age-matched *Aqp5*^*−/−*^ and *Aqp5*^*+/+*^ mice. Interestingly, histological examination using H&E staining revealed a higher abundance of lipid droplets and more pronounced vacuolar degeneration in the regenerating liver of *Aqp5*^*−/−*^ mice compared to *Aqp5*^*+/+*^ mice at 48 and 72 h post-PHx (Fig. 2*A*). Furthermore, the liver-to-body weight ratio was significantly reduced in *Aqp5*^*−/−*^ mice compared to their wild-type counterparts at 36, 48, and 72 h after PHx (Fig. 2*B*). Moreover, the survival rate of *Aqp5*^*−/−*^ mice was significantly lower compared to that of *Aqp5*^*+/+*^ mice (Fig. S2*B*). Additionally, at 14 days post-PHx, the liver-to-body weight ratio in *Aqp5*^*−/−*^ mice were also found to be lower than that in *Aqp5*^*+/+*^ mice (Fig. S2*C*). Additionally, serum levels of AST and ALT were markedly elevated at these time points in *Aqp5*^*−/−*^ mice (Fig. 2*C*).

After 36, 48, and 72 h post-PHx, a reduced number of Ki67-positive hepatocytes were observed in the livers of *Aqp5*^*−/−*^ mice compared to *Aqp5*^*+/+*^ mice (Fig. 2*D*). The expression level of ki67 reached its peak at 48 h after PHx; however, the number of positive cells in *Aqp5*^*−/−*^ mice was only one-third that of *Aqp5*^*+/+*^ mice (Fig. 2*E*). Western blot analysis revealed that *Aqp5*^*+/+*^ mice showed significant proliferation 36 h after 70% PHx, while *Aqp5*^*−/−*^ mice showed significant proliferation 48 h later. Liver regeneration was delayed in *Aqp5*^*−/−*^ mice compared to *Aqp5*^*+/+*^ mice. (Fig. 2, *F* and *G*).

### *Aqp5* deficiency resulted in redox imbalance and lipid droplet accumulation during liver regeneration

**Figure 3:**
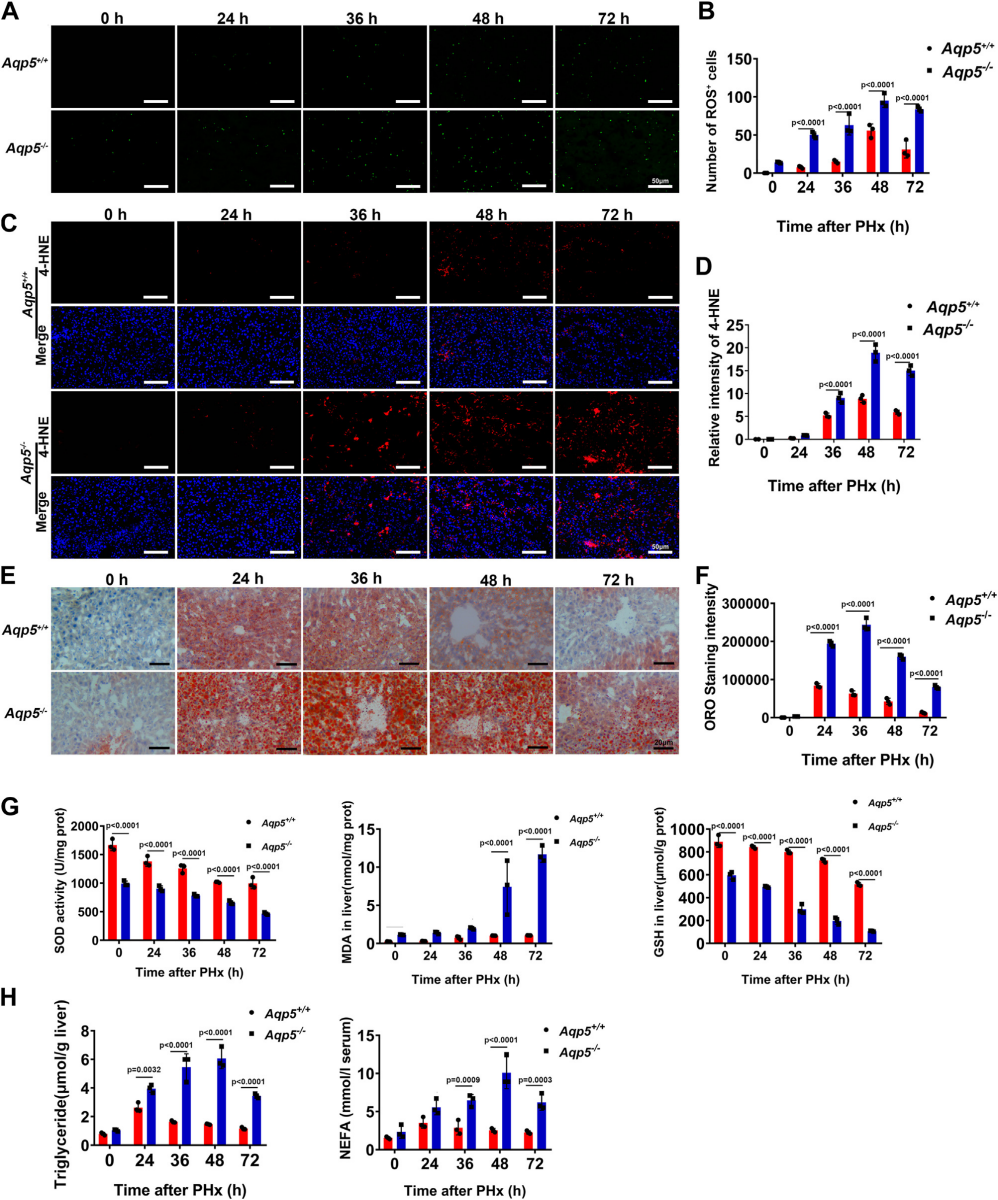
***Aqp5* deficiency resulted in lipid droplet accumulation and redox imbalance during liver regeneration.***A*, ROS in *Aqp5*^*−/−*^ and *Aqp5*^*+/+*^ mice liver at indicated time points after 70% PHx. *B*, ROS positive hepatocytes were calculated. For a 2-way ANOVA comparing time points (0, 24, 36, 48, 72 h) and genotype (wild-type *versus* knockout) for their effect on number of ROS^+^cells: Time: F (4, 20) = 84.95, *p* < 0.0001; Genotype: F (1, 20) = 237.9, *p* < 0.0001; Interaction (Time × Genotype): F (4, 20) = 6.973, *p* = 0.0011. *C*, 4-HNE staining in *Aqp5*^*−/−*^ and *Aqp5*^*+/+*^ mice liver at indicated time points after PHx. *D*, 4-HNE staining intensity analyzed by ImageJ software. For a 2-way ANOVA comparing time points (0, 24, 36, 48, 72 h) and genotype (wild-type vs. knockout) for their effect on relative intensity of 4-HNE: Time: F (4, 19) = 290.0, *p* < 0.0001; Genotype: F (1, 19) = 220.3, *p* < 0.0001; Interaction (Time × Genotype): F (4, 20) = 42.86, *p* < 0.0001. *E*, ORO staining was performed at indicated time points after PHx. *F*, the quantitative analysis of ORO staining by ImageJ software. For a 2-way ANOVA comparing time points (0, 24, 36, 48, 72 h) and genotype (wild-type vs. knockout) for their effect on ORO Staining intensity: Time: F (4, 20) = 474.0, *p* < 0.0001; Genotype: F (1, 20) = 1333, *p* < 0.0001; Interaction (Time × Genotype): F (4, 20) = 126.1, *p* < 0.0001. *G*, alteration of GSH, SOD, and MDA in *Aqp5*^*−/−*^ and *Aqp5*^*+/+*^ liver at indicated time points after PHx. For a 2-way ANOVA comparing time points (0, 24, 36, 48, 72 h) and genotype (wild-type *versus* knockout) for their effect on SOD: Time: F (4, 20) = 89.60, *p* < 0.0001; Genotype: F (1, 20) = 502.2, *p* < 0.0001; Interaction (Time × Genotype): F (4, 20) = 5.196, *p* = 0.0049. For a 2-way ANOVA comparing time points (0, 24, 36, 48, 72 h) and genotype (wild-type *versus* knockout) for their effect on MDA: Time: F (4, 20) = 27.21, *p* < 0.0001; Genotype: F (1, 20) = 89.06, *p* < 0.0001; Interaction (Time × Genotype): F (4, 20) = 20.57, *p* < 0.0001. For a 2-way ANOVA comparing time points (0, 24, 36, 48, 72 h) and genotype (wild-type *versus* knockout) for their effect on GSH: Time: F (4, 20) = 240.2, *p* < 0.0001; Genotype: F (1, 20) = 1803, *p* < 0.0001; Interaction (Time × Genotype): F (4, 20) = 20.57, *p* < 0.0001. *H*, hepatic TG were evaluated at indicated time points after PHx. Serum NEFA were evaluated at indicated time points after PHx. For a 2-way ANOVA comparing time points (0, 24, 36, 48, 72 h) and genotype (wild-type *versus* knockout) for their effect on TG: Time: F (4, 20) = 52.07, *p* < 0.0001; Genotype: F (1, 20) = 282.0, *p* < 0.0001; Interaction (Time × Genotype): F (4, 20) = 29.80, *p* < 0.0001. For a 2-way ANOVA comparing time points (0, 24, 36, 48, 72 h) and genotype (wild-type *versus* knockout) for their effect on NEFA: Time: F (4, 20) = 15.98, *p* < 0.0001; Genotype: F (1, 20) = 104.8, *p* < 0.0001; Interaction (Time × Genotype): F (4, 20) = 10.62, *p* < 0.0001. All statistics were calculated by using Two-way ANOVA with Sidak's multiple comparison test. Data were shown as mean ± SD. Scale bar: (*A* and *C*) 50 μm. (*E*) 20 μm.

The ROS staining results demonstrated a significant elevation of ROS levels in the liver of *Aqp5*^*−/−*^ mice at 0, 24, 36, 48, and 72 h post-PHx (Fig. 3*A*). Specifically, the number of ROS-positive cells rose from 55.67 ± 8.74 in *Aqp5*^*+/+*^ mice to 95 ± 8.89 in *Aqp5*^*−/−*^ mice at the time point of 48 h after PHx (*p* < 0.01; n = 3 per group), and from 31 ± 11.36 in *Aqp5*^*+/+*^ mice to 83.67 ± 3.06 in *Aqp5*^*−/−*^ mice at 72 h after PHx (*p* < 0.01; n = 3 per group) (Fig. 3*B*). The immunohistochemical staining of 4-HNE demonstrated an accumulation of lipid peroxidation end-products in the liver of *Aqp5*^*−/−*^ mice throughout the proliferative phase of regeneration at 24, 36, 48, and 72 h after PHx (Fig. 3*C*). Importantly, there was a significant increase in the intensity of 4-HNE from 8.847 ± 0.82 in *Aqp5*^*+/+*^ mice to 18.9 ± 1.85 in *Aqp5*^*−/−*^ mice at 48 h after PHx (*p* < 0.01; n = 3 per group), and from 5.91 ± 0.43 in *Aqp5*^*+/+*^ mice to 15 ± 1.11 in *Aqp5*^*−/−*^ mice at 72 h after PHx (*p* < 0.01; n = 3 per group) (Fig. 3*D*). Meanwhile, ORO revealed hepatic lipid accumulation in the liver of *Aqp5*^*−/−*^ mice throughout the proliferative phase of regeneration at 0, 24, 36, 48, and 72 h after PHx (Fig. 3*E*). The intensity of neutral TG significantly increased from 42,579 ± 8312 in *Aqp5*^*+/+*^ mice to 160,053 ± 6648 in *Aqp5*^*−/−*^ mice at 48 h after PHx (*p* < 0.01; n = 3 per group), and from 12,870 ± 2011 in *Aqp5*^*+/+*^ mice to 79,793 ± 4427 in *Aqp5*^*−/−*^ mice at 72h after PHx (*p* < 0.01; n = 3 per group) (Fig. 3*F*). Furthermore, the biochemical analysis revealed a significant elevation in MDA levels and reduction in SOD and GSH levels in *Aqp5*^*−/−*^ regenerating liver compared to *Aqp5*^*+/+*^ at 48 and 72 h after PHx (Fig. 3*G*). Interestingly, there was an increased transient hepatic accumulation of TG in *Aqp5*^*−/−*^ mice during the 72-h period following PHx, accompanied by elevated serum NEFA levels at the same time points (Fig. 3*H*).

### The PPAR pathway was suppressed during hepatic regeneration in *Aqp5*^*−/−*^ mice

We identified a total of 16,599 genes in the livers of *Aqp5*^*+/+*^ and *Aqp5*^*−/−*^ mice at 72 h post 70% PHx using RNA-seq analysis. Among these genes, there were 770 differentially expressed genes, including 247 downregulated and 523 upregulated genes.

**Figure 4:**
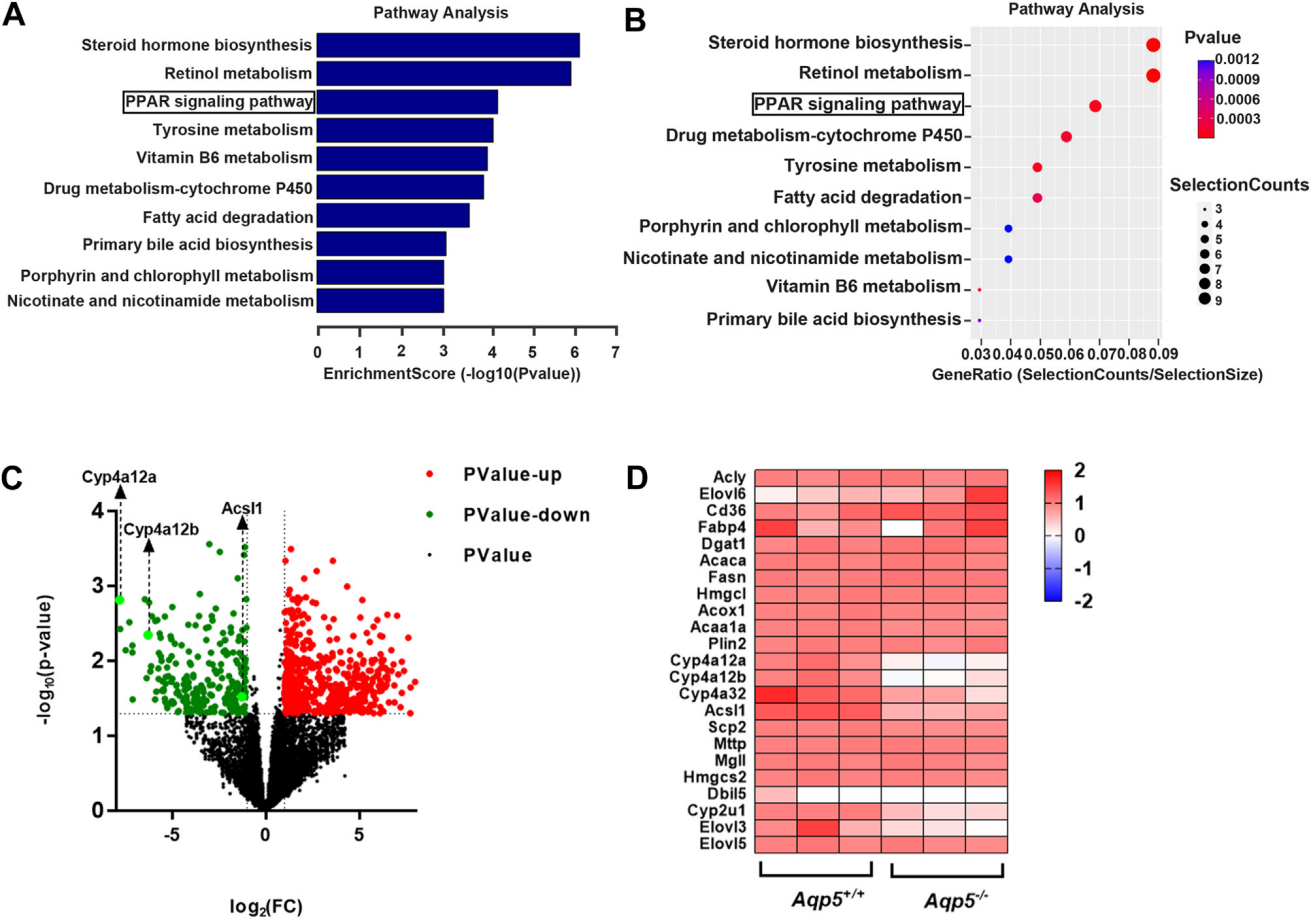
***Aqp5* deficiency affected the expression of the β-oxidative signaling pathway and related genes.***A*, GO analysis and KEGG pathway analysis of downregulated mRNA between *Aqp5*^*+/+*^ and *Aqp5*^*−/−*^ mice 72h after PHx. *B*, relevant pathways were identified for downregulated mRNA between *Aqp5*^*+/+*^ and *Aqp5*^*−/−*^ mice 72 h after PHx. *C*, Volcano plot of target genes between *Aqp5*^*+/+*^ and *Aqp5*^*−/−*^ mice 72h after PHx screened by RNA-sequencing. ACSL1, Cyp4a12a and Cyp4a12b are decreased in content (*Aqp5*^*−/−*^*versus Aqp5*^*+/+*^). *D*, Heatmap of target genes between *Aqp5*^*+/+*^ and *Aqp5*^*−/−*^ mice 72 h after PHx screened by RNA-sequencing.

To obtain a comprehensive understanding of the signaling pathways underlying lipid metabolism in the differentially expressed genes identified in *Aqp5*^*−/−*^ mice at 72 h after PHx, we performed pathway enrichment analyses using Gene Ontology (GO) and the Kyoto Encyclopedia of Genes and Genomes (KEGG). Our KEGG analysis revealed a significant association between the downregulated PPAR pathway and lipid metabolism (Fig. 4, *A* and *B*). The differential gene expressions are visually represented in a volcano plot, where it is worth noting that significant changes were observed in key downstream target genes associated with lipid metabolism in the PPAR pathway within the downregulated gene group, namely ACSL1, Cyp4a12a, and Cyp4a12b (Figs. 4*C* and S2*D*). In contrast to the gene expression profile observed in *Aqp5*^*+/+*^ mice after PHx, distinct patterns of differentially expressed genes related to lipid metabolism were exhibited by *Aqp5*^*−/−*^ mice as depicted by a heatmap analysis (Fig. 4*D*).

**Figure 5:**
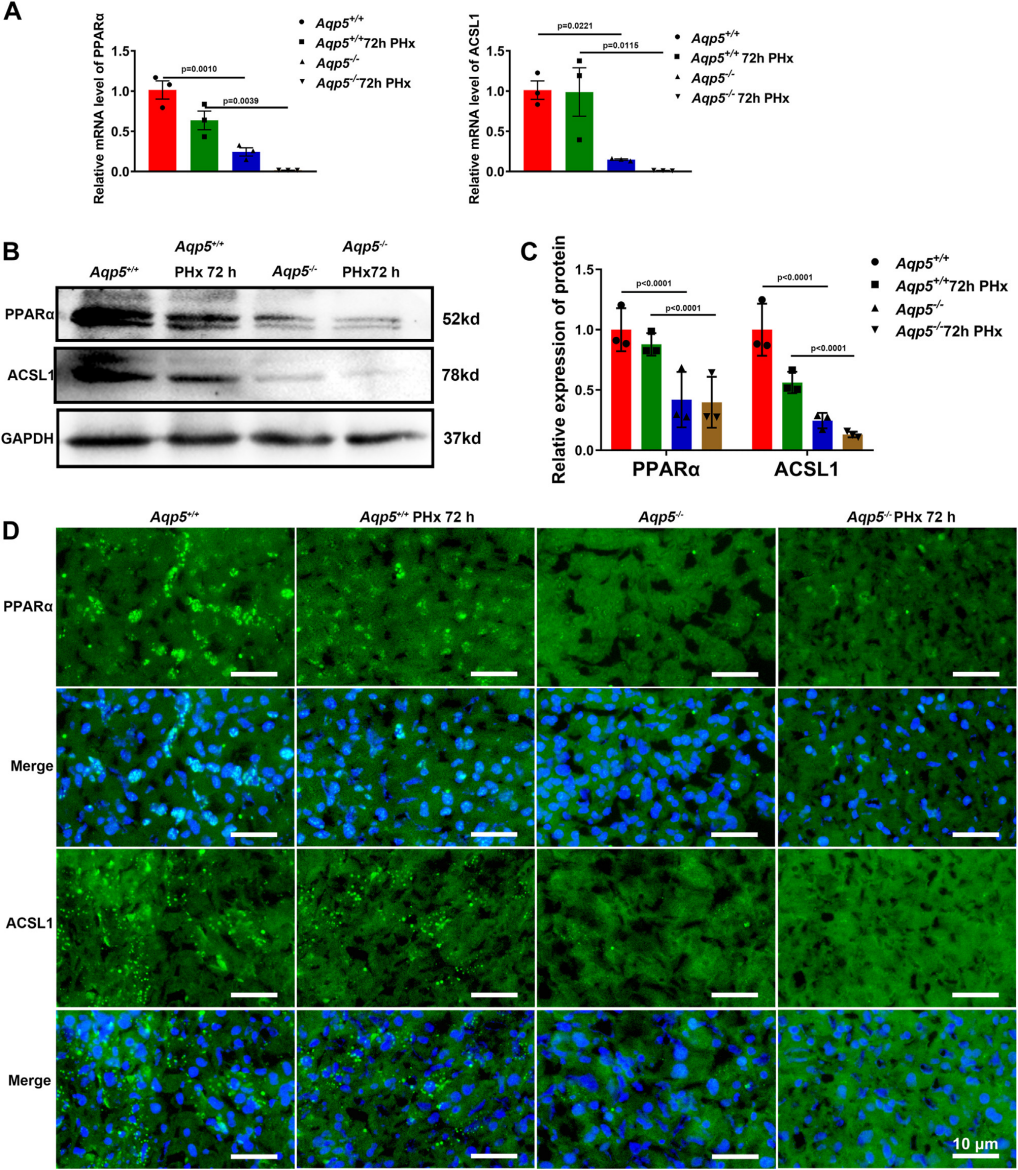
**The PPAR pathway was decreased in *Aqp5***^***−/−***^**liver during liver regeneration.***A*, mRNA expression of PPARα and ACSL1 at 0h and 72 h after PHx was determined by qPCR (n = 3 mice/group). Statistics were calculated by using one-way ANOVA with Sidak's multiple comparison test. The PPARα results showed that the overall difference is significant, with F (3, 8) = 26.60, *p* = 0.0002. The ACSL1 results showed that the overall difference is significant, with F (3, 8) = 10.98, *p* = 0.0033. *B*, Western blotting of PPARα, ACSL1, and GAPDH at 0h and 72 h after PHx (n = 3). *C*, expression of PPARα and ACSL1 was quantified. Statistics were calculated by using one-way ANOVA with Sidak's multiple comparison test. The PPARα results showed that the overall difference is significant, with F (3, 8) = 196.4, *p* < 0.0001. The ACSL1 results showed that the overall difference is significant, with F (3, 8) = 330.8, *p* < 0.0001. *D*, immunofluorescent staining of PPARα and ACSL1 at 0 h and 72 h after PHx. Nucleus were counterstained with DAPI. Data were shown as mean ± SD. Scale bar: (*D*) 10 μm.

The liver tissues from both *Aqp5*^*+/+*^ and *Aqp5*^*−/−*^ mice were examined 72 h after PHx. RT-qPCR assays revealed a significant downregulation of hepatic PPARα and ACSL1 in *Aqp5*^*−/−*^ mice compared to *Aqp5*^*+/+*^ mice (Fig. 5*A*). The expression of the key genes for lipid oxidation, CPT1α and CPT2, was also decreased (Fig. S2*E*). Similarly, Western blot assay demonstrated a significant decrease in hepatic PPARα and ACSL1 levels in *Aqp5*^*−/−*^ mice compared to *Aqp5*^*+/+*^ mice (Fig. 5, *B* and *C*). The immunofluorescent staining revealed a decrease in the nuclear localization of PPARα and ACSL1 in the regenerating liver of *Aqp5*^*−/−*^ mice at 72 h post PHx, compared to *Aqp5*^*+/+*^ mice (Fig. 5*D*). The primary hepatocytes were treated with lentivirus for *Aqp5* overexpression and knock-down, followed by subsequent measurement of PPARα and ACSL1 levels (Fig. S2, *F*–*I*).

### The administration of WY-14643 resulted in a reduction in lipid accumulation and an improvement in liver regeneration in *Aqp5*^*−/−*^ mice

**Figure 6:**
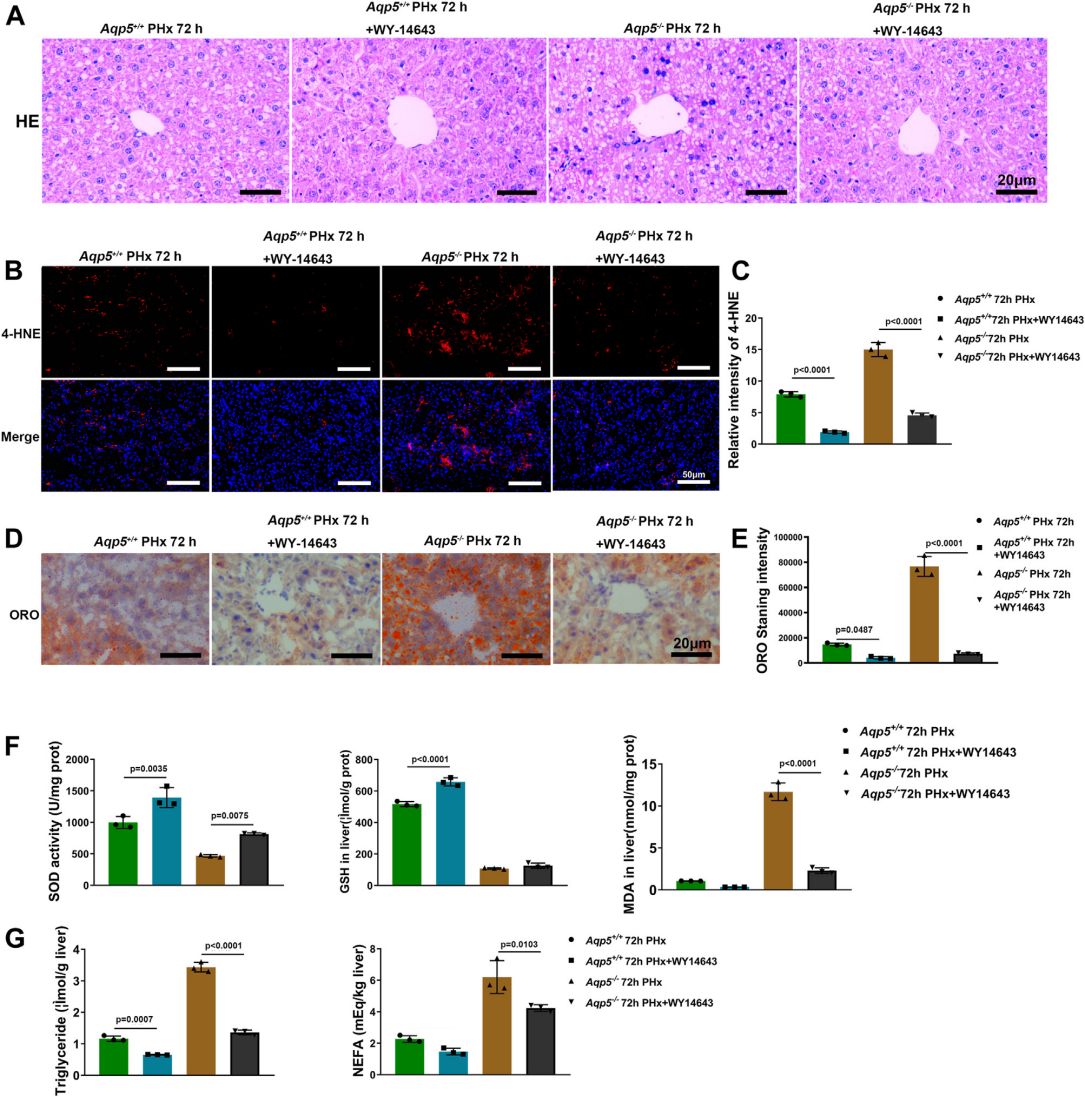
**WY-14643 reduced lipid accumulation and improved liver regeneration in *Aqp5***^***−/−***^**mice.***A*, H&E staining was performed at 72 h and 72 h + WY-14643 after PHx. *B*, 4-HNE staining in *Aqp5*^*−/−*^ and *Aqp5*^*+/+*^ mice liver at 72 h and 72 h + WY-14643 after PHx. *C*, 4-HNE staining intensity analyzed by ImageJ software. Statistics were calculated by using one-way ANOVA with Sidak's multiple comparison test. The results showed that the overall difference is significant, with F (3, 8) = 243.9, *p* < 0.0001. *D*, ORO staining was performed at 72h and 72 h + WY-14643 after PHx. *E*, The quantitative analysis of ORO staining by ImageJ software. Statistics were calculated by using one-way ANOVA with Sidak's multiple comparison test. The results showed that the overall difference is significant, with F (3, 8) = 219.0, *p* < 0.0001. *F*, alteration of GSH, SOD, and MDA in *Aqp5*^*−/−*^ and *Aqp5*^*+/+*^ liver at 72 h and 72 h + WY-14643 after PHx. Statistics were calculated by using one-way ANOVA with Sidak's multiple comparison test. The SOD results showed that the overall difference is significant, with F (3, 8) = 52.16, *p* < 0.0001. The GSH results showed that the overall difference is significant, with F (3, 8) = 762.0, *p* < 0.0001. The MDA results showed that the overall difference is significant, with F (3, 8) = 273.0, *p* < 0.0001. *G*, hepatic TG were evaluated at 72h and 72h + WY-14643 after PHx. Serum NEFA were evaluated at 72h and 72h + WY-14643 after PHx. Statistics were calculated by using one-way ANOVA with Sidak's multiple comparison test. The TG results showed that the overall difference is significant, with F (3, 8) = 504.1, *p* < 0.0001. The NEFA results showed that the overall difference is significant, with F (3, 8) = 44.18, *p* < 0.0001. Data were shown as mean ± SD. Scale bar: (*A* and *D*) 20 μm. (*B*) 50 μm.

To investigate the potential role of lipid accumulation in impaired liver regeneration, we administered WY-14643, a PPAR pathway agonist, to both *Aqp5*^*+/+*^ and *Aqp5*^*−/−*^ mice. The H&E staining revealed a significant decrease in lipid droplets and vacuolar degeneration in the regenerating liver of *Aqp5*^*−/−*^ + WY-14643 mice compared to *Aqp5*^*−/−*^ mice at 72 h post-PHx (Fig. 6*A*). Additionally, the 4-HNE staining revealed a significant reduction in the accumulation of lipid peroxidation end-products within hepatocytes of mice at 72 h after PHx following WY-14643 treatment (Fig. 6*B*). The intensity of 4-HNE exhibited a substantial decrease from 15 ± 1.11 in *Aqp5*^*−/−*^ mice to 4.576 ± 0.37 in *Aqp5*^*−/−*^ + WY-14643 mice at 72 h after PHx (*p* < 0.001, n = 3 per group). (Fig. 6*C*). Additionally, the ORO staining revealed a significant reduction in the accumulation of neutral TG within hepatocytes of mice at 72 h after PHx following WY-14643 treatment (Fig. 6*D*). The intensity of neutral triglycerides decreased significantly from 76,705 ± 7868 in *Aqp5*^*−/−*^ mice to 7352 ± 673.1 in *Aqp5*^*−/−*^+ WY-14643 mice (*p* < 0.001, n = 3 per group) (Fig. 6*E*). Furthermore, biochemical analysis revealed that treatment with WY-14643 led to a significant decrease in MDA levels and an elevation in SOD and GSH levels in the regenerating liver of both *Aqp5*^*+/+*^ and *Aqp5*^*−/−*^ mice during the 72-h period following PHx (Fig. 6*F*). Moreover, WY-14643 treatment effectively decreased hepatic tissue TG and serum NEFA levels in both *Aqp5*^*+/+*^ and *Aqp5*^*−/−*^ mice 72 h after PHx (Fig. 6*G*).

### The PPAR pathway was activated by WY-14643, leading to the restoration of hepatocyte proliferation in *Aqp5*^*−/−*^ mice

**Figure 7:**
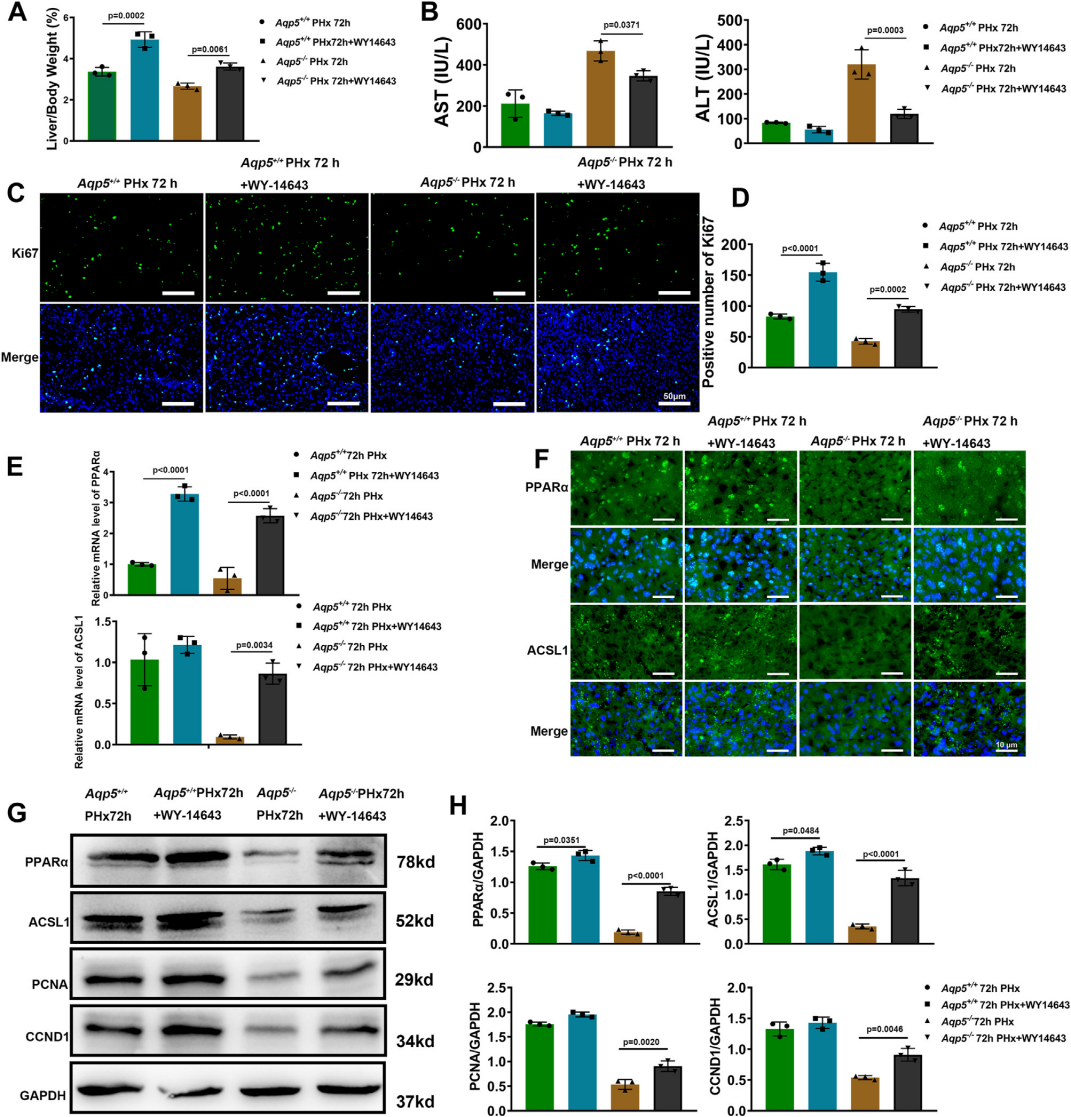
**WY-14643 directly activated PPAR pathway and restored hepatocyte proliferation in *Aqp5***^***−/−***^**mice.***A*, Liver-to-body weight ratios were calculated at 72 h and 72 h + WY-14643 after PHx. Statistics were calculated by using one-way ANOVA with Sidak's multiple comparison test. The results showed that the overall difference is significant, with F (3, 8) = 44.76, *p* < 0.0001. *B*, the activity of AST and ALT was measured in serum of *Aqp5*^*−/−*^ mice and *Aqp5*^*+/+*^ mice at 72h and 72h + WY-14643 after PHx. Statistics were calculated by using one-way ANOVA with Sidak's multiple comparison test. The AST results showed that the overall difference is significant, with F (3, 8) = 29.82, *p* = 0.0001. The ALT results showed that the overall difference is significant, with F (3, 8) = 42.38, *p* < 0.0001. *C*, immunofluorescence of Ki67 was performed at 72 h and 72 h + WY-14643 after PHx. *D*, Ki67 positive hepatocytes were calculated. Statistics were calculated by using one-way ANOVA with Sidak's multiple comparison test. The results showed that the overall difference is significant, with F (3, 8) = 95.71, *p* < 0.0001. *E*, mRNA expression of PPARα and ACSL1 at 72 h and 72 h + WY-14643 after PHx was determined by qPCR. Statistics were calculated by using one-way ANOVA with Sidak's multiple comparison test. The PPARα results showed that the overall difference is significant, with F (3, 8) = 85.09, *p* < 0.0001. The ACSL1 results showed that the overall difference is significant, with F (3, 8) = 22.64, *p* = 0.0003. *F*, Immunofluorescent staining of PPARα and ACSL1 at 72 h and 72 h + WY-14643 after PHx. Nuclei were counterstained with DAPI. *G*, Western blotting of PPARα, ACSL1, PCNA, CCND1, and GAPDH at 72h and 72h + WY-14643 after PHx. *H*, PPARα, ACSL1, PCNA, and CCND1 expression were quantified (n = 3). Statistics were calculated by using one-way ANOVA with Sidak's multiple comparison test. The PPARα results showed that the overall difference is significant, with F (3, 8) = 238.8, *p* < 0.0001. The ACSL1 results showed that the overall difference is significant, with F (3, 8) = 124.1, *p* < 0.0001. The PCNA results showed that the overall difference is significant, with F (3, 8) = 219.2, *p* < 0.0001. The CCND1 results showed that the overall difference is significant, with F (3, 8) = 60.93, *p* < 0.0001. Data were shown as mean ± SD. ∗*p* < 0.05; ∗∗*p* < 0.01; ∗∗∗*p* < 0.001. Scale bar: (*C*) 50 μm. (*F*) 10 μm.

The liver-to-body weight ratio exhibited a significant increase at 72 h after PHx in WY-14643-treated mice (*p* < 0.01) (Fig. 7*A*). Additionally, the serum levels of AST and ALT showed a significant decrease at 72 h after PHx in *Aqp5*^*−/−*^ + WY-14643 mice (Fig. 7*B*). The number of Ki67-positive hepatocytes in *Aqp5*^*−/−*^ + WY-14643 mice at 72 h post-PHx was significantly higher compared to that in *Aqp5*^*−/−*^ mice (Fig. 7*C*). Specifically, the count of Ki67-positive cells increased from 42.66 ± 4.51 in the *Aqp5*^*−/−*^ mice to 94.66 ± 4.51 in the *Aqp5*^*−/−*^ + WY-14643 mice (*p* < 0.01; n = 3 per group) (Fig. 7*D*). The RT-qPCR assays revealed a significant upregulation of hepatic PPARα and ACSL1 in *Aqp5*^*−/−*^ + WY-14643 mice compared to *Aqp5*^*−/−*^ at 72 h after PHx (Fig. 7*E*). Immunofluorescent staining demonstrated an enhanced nuclear localization of PPARα and ACSL1 in the regenerating liver of *Aqp5*^*−/−*^ + WY-14643 mice at 72 h after PHx, as compared to *Aqp5*^*−/−*^ mice (Fig. 7*F*). The Western blot assays revealed a significant upregulation of PPARα, ACSL1, PCNA, and CCND1 levels in *Aqp5*^*−/−*^ + WY-14643 mice at 72 h post PHx compared to *Aqp5*^*−/−*^mice (Fig. 7, *G* and *H*).

We infected primary hepatocytes with lentivirus to disrupt *Aqp5* expression and subsequently treated them with ROS scavenger (NAC) and PPAR agonist (WY-14643) for 24 h to measure ROS levels. It was observed that the level of ROS increased in cells affected by *Aqp5* interference, while both NAC and WY-14643 decreased the ROS level. Given that Aqp5 is located on the cell membrane and facilitates the transport of water and hydrogen peroxide, we hypothesize that *Aqp5* primarily influences hydrogen peroxide transport. Therefore, we employed a hydrogen peroxide probe to assess the impact of different levels of Aqp5 expression on H_2_O_2_ transportation. Relevant data are presented in the Fig. S3, *A*–*D*. The experimental findings demonstrate that *Aqp5* predominantly affects H_2_O_2_ transport, consequently leading to intracellular oxidative stress.

## Discussion

Referred to as the primary clinical intervention for liver diseases, especially liver cancers, partial hepatectomy (PHx) is currently employed (42, 43).

However, liver damage caused by exposure to harmful chemicals, viral infections, chronic inflammation, or excessive alcohol consumption can impede the natural process of liver regeneration and lead to severe hepatic failure with associated morbidity and mortality (44, 45). As a result, it is imperative that we gain a comprehensive understanding of the molecular and cellular mechanisms that govern the regenerative capacity of the liver following partial hepatectomy.

The process of liver regeneration following PHx is a complex and multi-level phenomenon involving priming, proliferation, and termination stages (46). The proliferative phase lasts for 48 h, during which hepatocytes enter the cell cycle and undergo significant proliferation (47). The intricate mechanism of liver regeneration remains a crucial focus in hepatic research (48, 49, 50). As Aqp5 is associated with multiple diseases in the liver (23, 25). In our study, we investigated the role of *Aqp5* in liver regeneration following major PHx. We demonstrated that depletion of *Aqp5* in hepatocytes significantly enhanced lipid accumulation and hindered liver regeneration post-PHx. The deficiency of *Aqp5* resulted in the downregulation of PPARα and ACSL1 expression, leading to a decrease in β-oxidation of fatty acids in the regenerating liver. Excess accumulation of H_2_O_2_ caused by Aqp5 deficiency may be responsible for this phenomenon.

Given that Aqp5 is located on the cell membrane and facilitates the transport of water and hydrogen peroxide, we hypothesize that *Aqp5* primarily influences hydrogen peroxide transport. Therefore, we employed a hydrogen peroxide probe to assess the impact of different levels of *Aqp5* expression on H_2_O_2_ transportation. Relevant data are presented in the Fig. S3, *E*–*H*. The experimental findings demonstrate that *Aqp5* predominantly affects H_2_O_2_ transport, consequently leading to intracellular oxidative stress.

The AQPs superfamily comprises multiple members that have been observed facilitating the diffusion of H_2_O_2_ across biological membranes (17). These specific AQPs, referred to as peroxiporins, are postulated to play crucial roles in regulating redox signaling in various organisms (16, 17, 18, 20). The aquaglyceroporin Aqp9 facilitates the translocation of water, glycerol, and other neutral solutes across the plasma membrane of hepatocytes adjacent to sinusoids (51, 52, 53). Additionally, Aqp9 serves as an efficient peroxiporin for transporting hydrogen peroxide (54). Research indicates that Aqp9 plays a crucial role in liver regeneration by modulating oxidative stress through its ability to transport H_2_O_2_, as evidenced by the reduced lipolysis observed after Aqp9 knockout during liver regeneration (21). Aqp5 is located in the mammalian plasma membrane and functions as an aquaporin, facilitating the transportation of water and other small molecules. Additionally, it acts as a peroxiporin responsible for transporting H_2_O_2_ (55, 56, 57). Our findings suggest that the deficiency of Aqp5 leads to significant intracellular accumulation of ROS, resulting in a downregulation of FAO. This study emphasizes the multifunctional role of Aqp5 as both a water channel and a protein involved in hydrogen peroxide metabolism, facilitating the cellular transport of water and hydrogen peroxide during the proliferative phase of liver regeneration.

Lipid accumulation is a physiological process observed during liver regeneration (58, 59, 60). The origin of accumulating lipid droplets during liver regeneration remains a subject of debate (61, 62, 63). Our findings indicate that *Aqp5* deficiency leads to a significant increase in lipid accumulation, suggesting distinct underlying mechanisms between pathological steatosis and lipid accumulation. In our study, as the proliferation phase progressed, there was a gradual reduction in lipid accumulation to normal levels within 48 to 72 h, indicating a diminishing reliance of liver regeneration on lipid accumulation. Concurrently, *Aqp5* knockouts suppressed FAO and exhibited decreased levels of β-oxidation compared to *Aqp5*^*+/+*^ mice. Consequently, we observed diminished expression levels of Ki67, PCNA, and CCND1 from 48 to 72 h post partial hepatectomy (PHx), ultimately resulting in reduced liver-to-body weight ratios due to the deficiency of *Aqp5*. In summary, the deletion of *Aqp5* delayed and impeded the utilization of lipid accumulation during the proliferative phase of liver regeneration.

When it comes to lipid metabolism, the formation of lipid droplets is initiated approximately 12 h after PHx, reaches its peak at 36 h, and subsequently declines to the basal level during the advanced stage of liver regeneration (64). Knockdown of systemic PPARα delays this process by 12 to 24 h, while deletion of liver-specific PPARα significantly retards hepatocyte proliferation by 32 h (65). The nuclear receptor PPARα is activated by ligands and plays a pivotal role in the regulation of lipid catabolism and energy homeostasis. Despite the indispensability of PPARα activation for liver regeneration, the underlying mechanisms remain elusive (31, 64, 65, 66, 67). The regulation of ACSL1 by PPARα exerts an influence on hepatic lipid metabolism (68). Hepatocyte fatty acid accumulation occurs in the absence of PPARα in the liver, leading to reduced levels of proteins and enzyme genes involved in fatty acid metabolism (such as ACSL1), thereby initiating and progressing liver disease (69). ACSL1 is the predominant isoform in the liver, accounting for 50% of total hepatic ACSL activity (70). The overexpression of ACSL1 enhances the proportion of oleic acid in diacylglycerol and phospholipids, thereby playing a crucial role in directing fatty acids towards triglyceride synthesis (71, 72). Additionally, excessive levels of free fatty acids can activate membrane transport through ACSL1, facilitating their efficient uptake into cells and subsequent deposition as triglycerides (73, 74, 75). After PHx, we observed a significant increase in TG and serum NEFA levels in the livers of *Aqp5*-deficient mice. Additionally, we utilized Oil Red O staining to predict the potential for delayed lipid accumulation, which was subsequently confirmed through other techniques such as 4-HNE staining and biochemical analyses. The presence of excessive lipids impairs DNA replication in hepatocytes (76). Therefore, *Aqp5*-mediated alteration of β-oxidation relies on the PPAR signaling pathway. Further investigation is warranted to explore the role of *Aqp5* as an inhibitor of lipid catabolism.

**Figure 8:**
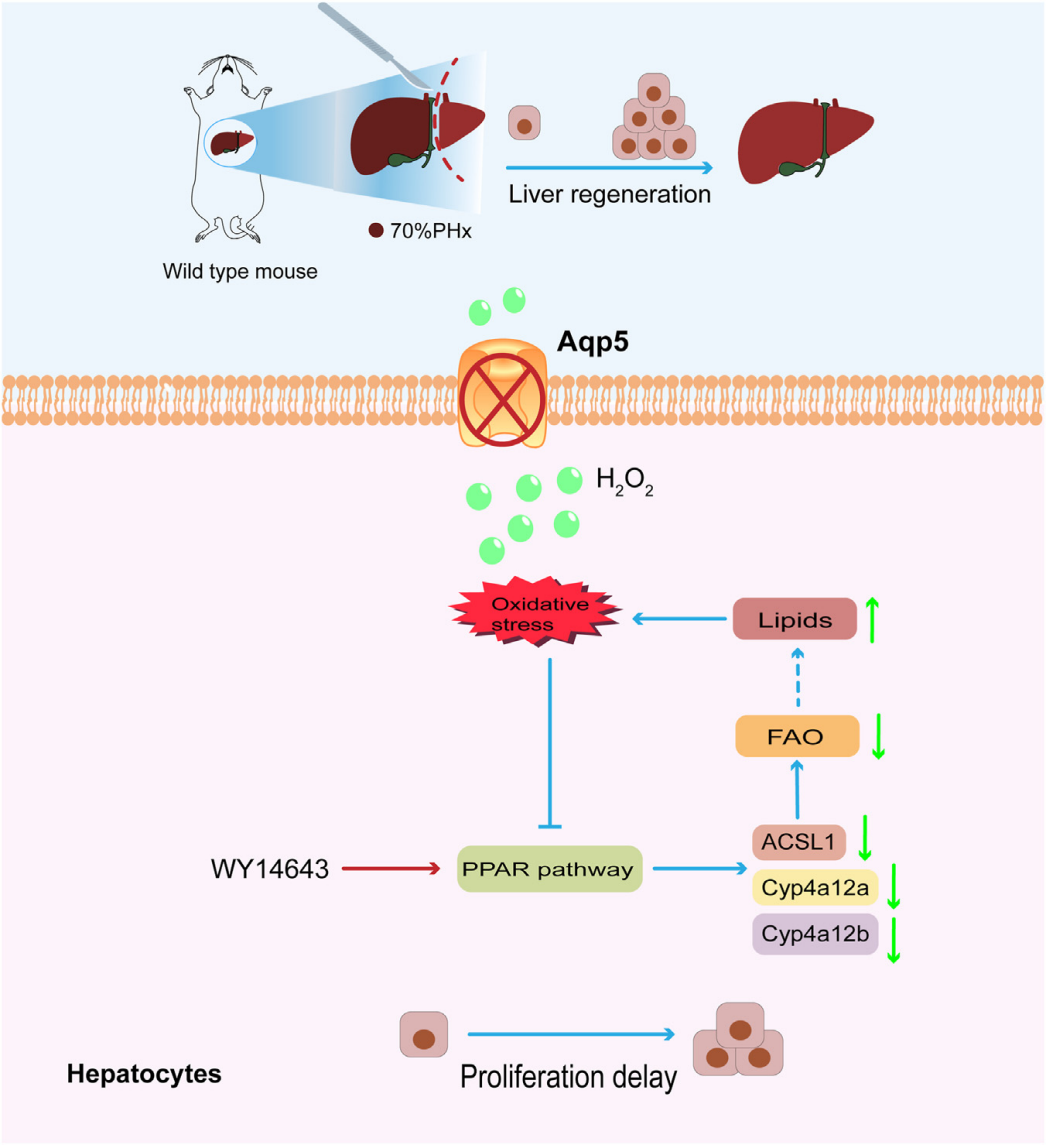
**Modes of the Aqp5/ROS/PPAR pathway axis leading to delay liver regeneration**.

A PPAR pathway agonist expedites hepatic regeneration and augments lipid metabolism (77). Previous studies have demonstrated that WY-14643, a PPAR pathway activator, significantly enhances liver size and proliferation during the process of liver regeneration (31). In conclusion, FAO plays a pivotal role in facilitating cell division during liver regeneration (78). In our study, we observed that administration of WY-14643 agonist targeting the PPAR pathway significantly attenuated delayed liver regeneration with improved hepatocyte proliferation while also reducing lipid accumulation caused by *Aqp5* deficiency during liver regeneration (Fig. 8).

## Experimental procedures

### Animals

Using CRISPR/Cas9 technology, *Aqp5*^*−/−*^ mice were produced by the high-flux electric transfer of fertilized eggs from Cyagen Biosciences Inc (Guangzhou, China). We used age-matched *Aqp5*^*+/+*^ and *Aqp5*^*−/−*^ mice for our study. The *Aqp5*^*−/−*^ and C57BL/6 mice were raised and bred in accordance with our previous studies (79). All procedures were performed in compliance with relevant laws and institutional guidelines and have been approved by the appropriate institutional committee(s). All testing was conducted under the approval of the Ethics Committee of the Medical College of Qingdao University (No.QDU-AEC-2023352) (No.QDU-HEC-2025035). The human studies that have been reported adhere to the principles of the Declaration of Helsinki.

For PHx, 3 month old male mice were induced with 4% isoflurane (RWD, Shenzhen, China) for anesthesia induction and maintained on 2%. A single ligature was utilized to excise the left lateral, left median, and right median lobes during PHx. To prevent postoperative hypothermia in conscious mice, supplemental heat was provided using temperature-controlled warming pads. Two weeks prior to PHx, intraperitoneal injection of the PPAR pathway agonist WY-14643 (TargetMol) was administered at a dose of 50 mg/kg/d.

### Biochemical analysis

Serum samples and supernatants from liver homogenates obtained from wild-type (*Aqp5*^*+/+*^) and *Aqp5*^*−/−*^ mice were prepared for quantitative detection using corresponding kits for nonesterified fatty acids (NEFA) (Nanjing Jiancheng Bioengineering Institute, A042-2-1) and triglycerides (TG) (Nanjing Jiancheng Bioengineering Institute, 110-2-1). Levels of serum aspartate transaminase (AST), serum alanine aminotransferase (ALT), superoxide dismutase (SOD), glutathione (GSH), and malondialdehyde (MDA) were determined using an automatic biochemical analyzer (Rayto Chemray800) in accordance with the manufacturer's instructions provided by Servicebio in Wuhan.

### Hematoxylin and Eosin (H&E) and immunofluorescence staining

Hematoxylin and Eosin staining was performed on 5 μm paraffin sections of hepatic tissue. Cryosections of hepatic tissue were fixed with 4% paraformaldehyde (Solarbio, Wuhan, China). Prior to incubation with secondary antibodies conjugated to fluorescein (1:200; Life Technologies), the cryosections underwent immunostaining for Aqp5 (1:200; Abcam), Ki-67 (1:200; Abcam, Cambridge, MA, USA), 4-HNE (1:200; Abcam), PPARα (1:200; Affinity Biosciences) and ACSL1 (1:200; ABclonal). The fluorescently labeled cryosections were visualized using a fluorescence microscope (Olympus, Tokyo Japan). The potential for non-specific staining can be effectively mitigated by employing isotype control antibodies that match the target antibody. If no signal was detected with the isotype control, this indicated high specificity of the target antibody.

### Oil Red O (ORO) staining

The hepatic tissue cryosections were fixed and subsequently coated with Oil Red O solution (Cyagen Biosciences Inc). Mayer's hematoxylin was utilized for the subsequent incubation of the cryosections. Gelatin glycerin (Servicebio) was applied to seal the cryosections. Positive density measurements were acquired using ImageJ software (National Institutes of Health).

### ROS activity assay

The ROS activity in hepatocytes was evaluated by employing DCFH-DA (2′,7′-dichlorodihydrofluorescein diacetate, Molecular Probes, Beyotime) staining. The samples were incubated with a ROS working solution, and the number of ROS-positive cells was quantified under a microscope.

### Quantitative real-time PCR

The isolation of total RNAs from liver tissues and their reverse transcription into cDNA were performed using a kit (TaKaRa) according to the manufacturer's instructions for quantitative real-time PCR. For relative quantification of PPARα (Forward Sequence: AACAACCCCCTTITTCATA; Reverse Sequence: GACGGTCTCCACGGACATG), ACSL1 (Forward Sequence: GAAGCCCAAGC CTCCAGAAC; Reverse Sequence: GCGATGAATGCACTCCGTTG), CPTα (Forward Sequence: CTTCCAACGCATGACAGCAC; Reverse Sequence: TTAA CCATGATCGGCCCTCG) and CPT2 (Forward Sequence: GAGGCATTTGTCA GGGAGCC; Reverse Sequence: CTGCTGCCAGATACCGTAGAG), the ChamQ SYBR qPCR master mix (Epizyme) was employed. GAPDH was selected as the invariant internal control for RT-qPCR and subsequent normalization. The comparative CT method was used to calculate relative expression levels.

### RNA sequencing and bioinformatics analysis

High-throughput transcriptome sequencing was performed by Cloud-Seq Biotech. In brief, liver tissue underwent total RNA extraction using TRIZOL reagent (Invitrogen). The Ribo-NEBNext rRNA Removal kit (Illumina) was utilized for the depletion of total RNA. Subsequently, an RNA library was generated using purified RNA samples and the TruSeq Stranded Total RNA Library Preparation kit (Illumina) following the manufacturer's instructions. The quality control and library quantification were conducted using a BioAnalyzer 2100 system. Subsequently, the denatured single-stranded DNA molecules from the 10-pM library were captured on Illumina flow cells and underwent *in situ* amplification and clustering. Finally, sequencing with 150 cycles was performed on an Illumina HiSeq sequencer following the manufacturer's protocol. Raw counts were generated using HTSeg software (v0.9.) and subsequently normalized by edgeR software.

### Western blot analysis

The liver tissue was dissected and proteins were extracted using RIPA buffer. Subsequently, the proteins were separated on SDS-PAGE gels and transferred onto PVDF membranes. The membranes were then incubated with antibodies against Aqp5 (1:1000; Abcam), PCNA (1:1000; Affinity Biosciences), CyclinD1 (1:1000; Affinity Biosciences), PPARα (1:1000; Affinity Biosciences), ACSL1 (1:1000; ABclonal), and GAPDH (1:3000; Kangchen Shanghai China). Afterwards, they were incubated with secondary fluorescein-conjugated antibodies (1:3000; Zhongshan Jianqiao Biotech). Image detection was performed using a Tannon imaging system (Tanon 5200).

### Hepatocytes isolation and culture

Primary hepatocytes were isolated from male C57BL/6J mice. Briefly, mice were anesthetized with isoflurane and the portal vein was cannulated with a 0.45 mm needle. Then the inferior vena cava (IVC) was immediately cut to allow fluid to drain. Hank's Balanced Salt Solution (HBSS; Solarbio) containing 5 mM glucose supplemented with 0.5 mM EGTA and 10 mM HEPES (pH 7.4 at 37 °C) was perfused. HBSS (Solarbio) containing Calcium and magnesium supplemented with 100 U/ml Penicillin and 0.1 mg/ml Streptomycin (Pen/Strep), 15 mM HEPES, and 100 U/ml of collagenase (Type IV, maokangbio) was then perfused to digest the liver. After sufficient digestion, the gall bladder was removed and the liver was excised and transferred to a dish containing 15 ml of the same medium used for digestion. Cells were liberated by tearing and shaking of the liver with forceps followed by gentle trituration. Then, the liver was dissolved in a residual digestive fluid and filtered with a 100 μm cell strainer. Hepatocytes were obtained by low-speed centrifugation (100 x g) for 2 min at 4˚C. Cell culture plates were coated with 0.03 mg/ml rat tail collagen I 2h in advance at room temperature and washed three times with sterile PBS before use. The cells were seeded onto 6-well or 96-well plates with 8 × 10^4^ cells/ml precoated with rat tail tendon collagen I (Shengyou Biotechnology Co., Ltd). Upon reaching a confluency of 70 to 80%, the cells were incubated with lentivirus, N-acetyl-L-cysteine (NAC) and PPAR pathway agonist WY-14643 (TargetMol), subsequently total protein were extracted for western blotting. Besides the cells were incubated with H_2_O_2_ (Sigma) and acetazolamide (AZ; MCE), inhibitors of Mammalian Aquaporin Water Channels.

### Transduction of recombinant lentivirus

To achieve the overexpression and knockdown of *Aqp5*, primary hepatocytes at passage two were seeded in cell culture flasks until they reached 30 to 50% confluency. Subsequently, cells were transfected with control, interfering, or overexpression lentiviral vectors using a commercially available kit (General Biol). Following a transfection period of 24 to 48 h, the culture medium was replaced with a fresh medium.

### Statistical analyses

The data were presented as mean ± standard deviation (SD). An unpaired two-tailed Student *t* test was employed to determine the differences between the two groups. For studies involving multiple tests, one- or two-way ANOVA was utilized. A significance level of *p* < 0.05 was considered statistically significant for each bilateral test (∗*p* < 0.05; ∗∗*p* < 0.01; ∗∗∗*p* < 0.001). GraphPad Prism software version 7 0.0 (GraphPad Software Inc.) was used for all statistical analyses.

## Data availability

The data of sequencing results for this study can be found in the GEO database (GSE284995).

Please see https://www.ncbi.nlm.nih.gov/geo/query/acc.cgi?acc=GSE284995 for more details.

## Supporting information

This article contains supporting information.

## Conflict of interest

The authors declare that they have no conflicts of interest with the contents of this article.

## Figures and Tables

**Supplementary Fig3:**