# Supplementary material for: Aquaporin five deficiency suppresses fatty acid oxidation and delays liver regeneration through the transcription factor PPAR
Source: J Biol Chem. 2025 Feb 11;301(3):108303. doi: 10.1016/j.jbc.2025.108303 (PMC11930093; doi:10.1016/j.jbc.2025.108303)
Supplement: Supplementary figure legend [file mmc1.docx]

**Supplementary figure legend**

**Supplementary Figure 1** (A) Immunofluorescence and immunohistochemical staining showed the expression of AQP5 in normal liver and Paracancerous tissues from human (red, green, AQP5; blue, DAPI). Scale bar: (A) 20μm.

**Supplementary Figure 2**  (A) Heatmap of target genes between *Aqp5^+/+^* and *Aqp5^−/−^* mice 72h after PHx screened by RNA-sequencing. (B) Survival of *Aqp5^+/+^* and *Aqp5^−/−^* mice (n = 15/group) after 70% PHx. (C) Liver to body weight ratio of *Aqp5^+/+^* and *Aqp5^−/−^* mice at indicated time points after 70% PHx (n = 12/group). Statistics were calculated by using Two-way ANOVA with Sidak’s multiple comparison test. For a 2-way ANOVA comparing time points (0, 1, 7, 14 days) and genotype (wild-type vs. knockout) for their effect on Liver / body weight : Time : F (3, 24) = 1344, p < 0.0001; Genotype : F (1, 24) = 66.36, p < 0.0001; Interaction (Time × Genotype) : F (3, 24) = 3.982, p < 0.0001. (D) mRNA expression of Cyp4a12a and Cyp4a12b at 72h after PHx was determined by qPCR. Statistics were calculated by using one-way ANOVA with Sidak’s multiple comparison test. The Cyp4a12a results showed that the overall difference is significant, with F (3, 8) = 53.13, p < 0.0001. The Cyp4a12b results showed that the overall difference is significant, with F (3, 8) = 49.77, p < 0.0001. (E) mRNA expression of CPT1α and CPT2 at 72h after PHx was determined by qPCR. Statistics were calculated by using one-way ANOVA with Sidak’s multiple comparison test. The CPT1α results showed that the overall difference is significant, with F (3, 8) = 117.6, p < 0.0001. The CPT2 results showed that the overall difference is significant, with F (3, 8) = 55.16, p < 0.0001. (F) Western blotting showed the expression level of *Aqp5*, PPAR-α, ACSL1 and β-actin in primary hepatocyte after *Aqp5* overexpression of lentivirus treatment. (G) *Aqp5*, PPAR-α, ACSL1 and β-actin expression was quantified. (n = 3). An unpaired two-tailed Student t-test was employed to determine the differences between the two groups. (H) Western blotting showed the expression level of *Aqp5*, PPAR-α, ACSL1 and β-actin in primary hepatocyte after the *Aqp5* knockdown lentivirus treatment. (I) *Aqp5*, PPAR-α, ACSL1 and β-actin expression was quantified. (n = 3) An unpaired two-tailed Student t-test was employed to determine the differences between the two groups. Data were shown as mean ± SD.

**Supplementary Figure 3** (A) ROS in primary hepatocytes after the *Aqp5* knockdown lentivirus and NAC treatment. (B) ROS staining intensity analyzed by ImageJ software. Statistics were calculated by using a One-way ANOVA with Sidak’s multiple comparisons. The results showed that the overall difference is significant, with F (2, 15) = 129.9, p < 0.0001. (C) ROS in primary hepatocytes after the *Aqp5* knockdown lentivirus and WY14647 treatment. (D) ROS staining intensity analyzed by ImageJ software. Statistics were calculated by using a One-way ANOVA with Sidak’s multiple comparisons. The results showed that the overall difference is significant, with F (2, 12) = 105.6, p < 0.0001. (E) H_2_O_2_ staining in primary hepatocytes after H_2_O_2_ Incubation and AZ treatment. (F) H_2_O_2_ staining intensity analyzed by ImageJ software. An unpaired two-tailed Student t-test was employed to determine the differences between the two groups. (G) H_2_O_2_ staining in primary hepatocytes after serum deprivation and AZ treatment. (H) H_2_O_2_ staining intensity analyzed by ImageJ software. An unpaired two-tailed Student t-test was employed to determine the differences between the two groups. Data were shown as mean ± SD. Scale bar: (A) (C) 25μm. (E) (G) 10μm.
